# Analysis of Carbon Nanoparticle Coatings via Wettability

**DOI:** 10.3390/nano14030301

**Published:** 2024-02-01

**Authors:** Raffaella Griffo, Francesco Di Natale, Mario Minale, Mariano Sirignano, Arianna Parisi, Claudia Carotenuto

**Affiliations:** 1 Dipartimento di Ingegneria, Università della Campania “L. Vanvitelli”, 81031 Aversa (Caserta), Italy; mario.minale@unicampania.it; 2 Dipartimento di Ingegneria Chimica, dei Materiali e della Produzione Industriale, Università di Napoli “Federico II”, 80125 Napoli, Italy; francesco.dinatale@unina.it (F.D.N.); mariano.sirignano@unina.it (M.S.); arianna.parisi@unina.it (A.P.)

**Keywords:** carbon nanoparticles, soot, thin film, wettability, superhydrophobic materials, contact angle

## Abstract

Wettability, typically estimated through the contact angle, is a fundamental property of surfaces with wide-ranging implications in both daily life and industrial processes. Recent scientific interest has been paid to the surfaces exhibiting extreme wettability: superhydrophobic and superhydrophilic surfaces, characterized by high water repellency and exceptional water wetting, respectively. Both chemical composition and morphology play a role in the determination of the wettability “performance” of a surface. To tune surface-wetting properties, we considered coatings of carbon nanoparticles (CNPs) in this study. They are a new class of nanomaterials synthesized in flames whose chemistry, dimension, and shape depend on combustion conditions. For the first time, we systematically studied the wettability of CNP coatings produced in a controlled rich ethylene/air flame stabilized over a McKenna burner. A selected substrate was intermittently inserted in the flame at 15 mm above the burner to form a thin coating thanks to a thermophoretic-driven deposition mechanism. The chemical-physical quality and the deposed quantity of the CNPs were varied by opportunely combing the substrate flame insertion number (from 1 to 256) and the carbon-to-oxygen ratio, C/O (from 0.67 to 0.87). The wettability of the coatings was evaluated by measuring the contact angle, CA, with the sessile drop method. When the C/O = 0.67, the CNPs were nearly spherical, smaller than 8 nm, and always generated hydrophilic coatings (CA < 35°). At higher C/O ratios, the CNPs reached dimensions of 100 nm, and fractal shape aggregates were formed. In this case, either hydrophilic (CA < 76°) or superhydrophobic (CA ~166°) behavior was observed, depending on the number of carbon nanoparticles deposed, i.e., film thickness. It is known that wettability is susceptible to liquid surface tension, and therefore, tests were conducted with different fluids to establish a correlation between the flame conditions and the nanostructure of the film. This method offers a fast and simple approach to determining mesoscale information for coating roughness and topographical homogeneity/inhomogeneity of their surfaces.

## 1. Introduction

Surface chemistry and texture regulate several of the technological properties of solids since they determine their interactions with the surrounding environment. Among them, wettability governs the interactions between a liquid and a solid surface [1]. By combining chemistry and texture, different degrees of wettability can be achieved, spanning from extremely wet surfaces to liquid-repellent ones. Nowadays, several scientific and technological efforts are dedicated to the study and production of surfaces with extreme wetting behaviors, with particular attention to water as a wetting liquid [2,3,4]. Superhydrophilic surfaces are useful for the manufacture of anti-fogging products, anti-biofouling medical devices and high-efficiency heat exchangers [5,6,7]; superhydrophilic surfaces exhibit anti-icing, anti-bacterial, and self-cleaning capabilities [8].

Recent developments in additive manufacturing and thin-film deposition techniques allowed the production of coatings with tuneable properties. In particular, a regular and tailored arrangement of nanometric structures of selected chemistries can be opportunely combined to reach customized wetting behaviors [9,10,11,12]. However, the production cost of regular nanostructured coatings renders their application feasible for high-added-value products but discourages their use in mass production. The manufacturing of regular nanostructured coatings is much easier for objects having simple, regular geometries, such as flat plates, cylinders, or spheres. For the large-scale production of objects with complex geometries, a non-regular coating is a more feasible option. This can be achieved via thin-film depositions in several ways, such as spin-coatings, chemical vapor deposition, surface impregnation, electrospray coating, etc. [13,14,15]. Typically, these are multi-step processes where submicronic or nanometric particles are previously dispersed in selected fluids and then deposed over the substrates.

Carbon-based particles produced in combustion processes have traditionally been considered an unwanted source of air pollution, but they have also been in commerce for more than a century, being an important ingredient in several everyday and industrial products. Among them, carbon black (CB) is one of the most largely produced particles, with successful applications in various sectors, including pigment production, battery technology, pneumatic systems, piping, cables, and more [16,17,18,19]. Carbon black is also largely used for coatings [20,21], mainly to enhance the conductivity and UV resistance of materials and to proficiently modify surface wettability. Typically, CB coatings are superhydrophobic [22,23]; however, few examples of the opposite superhydrophilic behavior [24] are reported. In recent years, other carbon-based particles have reverted their reputation as pollutants, becoming new “smart” materials with unconventional chemical-physical properties. The most well-known examples include carbon nanotubes and graphene. Carbon nanoparticles (CNPs) are emerging as a nanomaterial with several interesting properties, which encourages their use in several sectors, such as medicine, sensors, optoelectronics, and energy storage [25,26,27,28,29]. CNPs can be synthesized in flames. This process is now gaining attention thanks to the new scientific understanding of particle formation mechanisms and the improvement of combustion control technologies.

During a combustion process, CNPs are formed according to a complex pathway of chemical reactions and physical interactions. Moving from the formation of the gas phase, polycyclic aromatic hydrocarbon (PAH) nucleation takes place, and the first particles are generated. These freshly nucleated domains are similar in chemical nature to the PAH they are constituted of, arranged in a spherical form, and with a loose or non-solid structure. Successively, these particles grow further, increasing their aromaticity, showing a lattice structure, and eventually forming fractal aggregates [30]. The latter are those generically intended as soot, whereas the generical term “CNP” comprises all types of particles formed in a flame. The type of fuel, fuel/oxidant ratio, and flame fluid dynamics affect the chemical-physical properties of the CNPs across the moiety described. The fine-tuning of the flame can be used to produce CNPs with different properties and in different quantities. To conveniently exploit these CNPs, they must be harvested from the flame. These can be deposited over a substrate directly inside the flame during the combustion process (one-step hot flame harvesting) or outside the flame by first capturing and then depositing them on a substrate (two-step cold flame harvesting) [31]. CNP deposition can be driven by pure hydrodynamics, essentially thermophoretic phenomena, or by adding external forces to enhance harvesting, e.g., electric fields [32]. The arrangement of CNPs over the collector surface is a complex phenomenon involving fluid dynamics and thermal and electromagnetic field profiles in flames and close to the substrate [32].

While the wettability of CB coatings has been largely explored [33], that of CNPs has not been analyzed in a systematic way. The few experimental data available so far indicate that the wettability of CNP coatings produced via flame synthesis significantly depends on the combustion process, which defines the CNP’s chemical-physical characteristics, and on the deposition mechanism, which determines their arrangement in the deposited film.

In this work, we produced CNP coatings using premixed laminar ethylene/air flames. In particular, various fuel-rich flames, from incipient to heavily soot ones, were used to produce and deposit different kinds of CNPs over a flat substrate to create chemically and structurally different coatings. A one-step hot flame harvesting procedure was selected using thermophoretic forces to deposit the CNPs from the hot flame to the cold substrate. After their production, the coated substrates were analyzed via wettability tests. The scope of this research activity was twofold: (i) to characterize the wettability of the CNP films produced in a flame and highlight their wettability performance, and (ii) to use the surface wettability analysis as a rapid and fine tool to test CNP coating features, including the spatial homogeneity/heterogeneity of the deposit obtained from different flame conditions. To achieve this goal, several liquids with different surface tensions were used for the wettability tests. The results obtained set the basis for the exploitation of these wettability tests as a standard method for characterizing films and coating morphologies. In fact, unlike other forms of thin-film morphology characterization (such as profilometry, SEM, and AFM), wettability has the advantage of being a mesoscale property that is measurable via rapid, economical, and facile methods requiring surfaces of a few mm².

The paper is structured in three main sections reporting: (i) a brief description of the principal theories on wettability for ideal and real surfaces; (ii) the detailed description of the lab-scale equipment used to produce the CNP coatings and of the experimental techniques adopted for their characterization; (iii) the presentation and discussion of the results. In the conclusions, all the outcomes of the research activities are drawn.

## 2. Wettability

**Figure 1:**
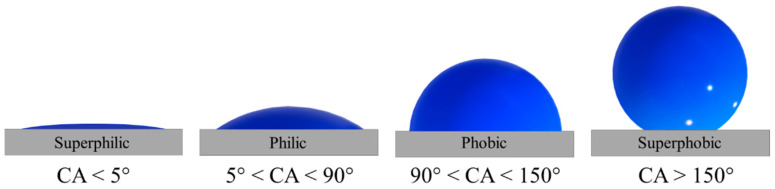
Sketch of a drop deposited on a solid surface with different wettability, from superphilic to superphobic.

Wettability is a physical property indicating the affinity of a liquid and a solid. Typically, it is evaluated by measuring the contact angle (CA) with the sessile drop method [34,35]. The CA is the angle formed between the solid surface and the line tangent to the drop profile in the three-phase point. Depending on the value assumed using the contact angle, four types of surfaces can be distinguished: superphilic, philic, phobic, and superphobic (Figure 1). In particular, if we refer to water, the terminology that identifies the surface wettability takes the prefix “hydro” [36].

The first correlation between the contact angle and interfacial properties dates back to Young (1805) [37,38], and it is related to a perfectly smooth and chemically homogeneous surface:

(1) $$cos\theta_{Y}=\frac{\gamma_{SV}-\gamma_{SL}}{\gamma_{LV}}$$

where $\gamma_{SV}$, $\gamma_{LV}$, and $\gamma_{SL}$, are the surface tensions for solid/vapor, liquid/vapor, and solid/liquid interfaces, respectively, and $\theta_{Y}$ is the intrinsic contact angle of a droplet on an ideal surface [39,40]. To date, the highest measured value of $\theta_{Y}$ is approximately 130° [41]. Young’s equation, Equation (1), cannot explain the contact angle values of a plethora of superhydrophobic surfaces with a CA significantly higher than 150° or oleophobic surfaces that repel liquids of very low surface tensions. The critical assumption in Young’s model is the perfectly smooth surface, which neglects all the information on surface texture, which has been identified as an indispensable characteristic for attaining special wetting properties in many biological and engineered materials [36].

Wenzel [42] and Cassie-Baxter [43] extended Young’s model to account for physical and chemical heterogeneities so as to describe the wettability of real surfaces. Wenzel’s model assumes that the liquid of the drops penetrates inside the asperities of the rough surface. The apparent contact angle, $\theta_{W}$, is thus related to the intrinsic Young’s contact angle through a roughness factor, *r*, defined as the ratio of the wetted to the projected area. As a consequence, under Wenzel’s model conditions, an increase in surface roughness directly amplifies the solid surface wettability:

(2) $$cos\theta_{W}=r\cdot cos\theta_{Y}$$

Conversely, Cassie-Baxter’s model assumes that the liquid of the drop does not penetrate the grooves of a rough surface, so bounded air pockets are created between the solid surface and the liquid. The apparent Cassie Baxter’s contact angle, $\theta_{CB}$, derives from an average of the contact angle between the solid and the liquid, $\theta_{Y},$ and that between the air pockets and the liquid, $\theta_{v}$:

(3) $$cos\theta_{CB}=f_{s}\cdot {cos\theta }_{Y}+f_{v}\cdot {cos\theta }_{v}$$

where $\theta_{Y}$ is Young’s contact angle, referring to a perfectly smooth solid surface, and $\theta_{v}$ is 180° since a drop of liquid suspended in air is perfectly spherical. The coefficients $f_{s}$ and $f_{v}$ are the area fractions of the liquid in contact with a solid and with the air pockets, respectively [44]. Since $f_{s}+f_{v}=1$, Equation (3) can be rewritten as follows:

(4) $$cos\theta_{CB}=f_{s}\cdot \left(cos\theta_{Y}+1\right)-1$$

A decrease in $f_{s}$, i.e., of the portion of solid wetted by the liquid, results in a decrease in wettability. According to Equations (3) and (4), it is possible for $\theta_{CB}$ to be significantly higher than 90° even if $\theta_{Y}$ is less than 90°. In other words, materials comprising hydrophilic smooth surfaces may lead to hydrophobic rough surfaces in the Cassie−Baxter state.

In the literature, a jungle of subscripts of *θ* is used [45] to discriminate among various types of contact angles, deriving from different measurement techniques, surface-averaging procedures, and wettability models. For the sake of simplicity, in the following paragraphs, we used the sole term contact angle, CA, to indicate the values measured in our experiments.

Finally, it is worth noticing that the CA value may vary over time. In certain situations, a wetting transition from a metastable non-wetting Cassie-Baxter state to a stable wetting Wenzel state might be observed [46,47,48]. The wetting transition [49] is an alteration in the wetting characteristics of the surface. This transition may occur spontaneously, such as under the effects of the evaporation or the gravitational force acting on the droplet, or it can be induced by various external stimuli, including vibrations, droplet bouncing, droplet compressions, and surface heterogeneities [46]. From a technological perspective, the wetting transition must be avoided, and a contact angle, stable over time, is a crucial asset in surface engineering.

## 3. Materials and Methods

### 3.1. Fabrication of the CNP Film

**Figure 2:**
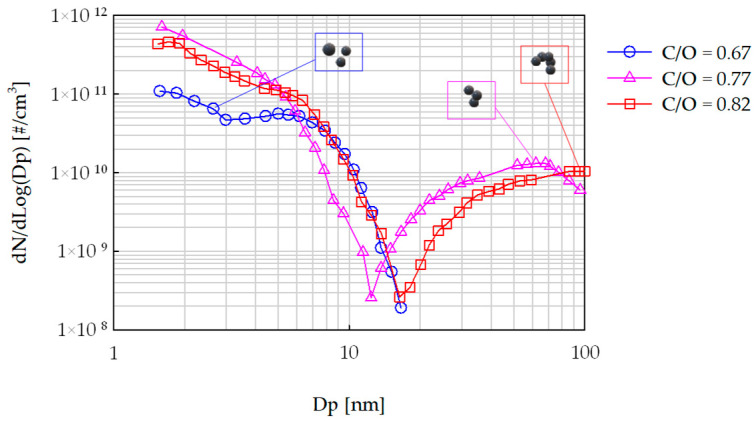
Particle size distributions measured at HAB = 15 mm in a laminar premixed ethylene-air flame under different C/O ratios [63].

A rich flat laminar premixed ethylene-air flame stabilized on a water-cooled McKenna burner was used to produce the CNPs [50,51]. The same lab-scale combustion unit was previously used for a CNP formation mechanism analysis [52,53,54] since it allows a complete characterization of the investigated flame conditions, including chemical-physical information on the produced particles. To favor soot and, more generically, CNP formation, rich flames with C/O ratios equal to 0.67, 0.77, 0.82, and 0.87 (corresponding to equivalence ratios of ϕ = 2.01, 2.31, 2.46, and 2.61, respectively) were used. The value of C/O = 0.67 is considered the incipient sooting limit [55], i.e., only small particles are formed and in low concentrations (approximately 0.1 ppm), while the flame with C/O = 0.87 is in a heavy sooting regime [56], i.e., large aggregates dominate CNP production with much higher concentrations (above 1 ppm). The CNPs were collected from the flame at a height above the burner (HAB) fixed at 15 mm, which can be considered the end of the flame where the CNP production reaches steady values. In former papers, state-of-the-art methodologies, such as differential mobility analysis (DMA), UV-Vis spectroscopy, atomic force microscopy (AFM), scanning tunneling microscopy (STM), size-exclusion chromatography (SEC), FT-IR, fluorescence spectroscopy, have been used to characterize the flame and the formed particles. Details on the physical-chemical properties of these CNPs are available in related papers [57,58,59,60,61,62]. For the relevance of this study, we show the particle size distributions (PSDs) of the CNPs produced at HAB = 15 mm with different C/O values (Figure 2).

The flame with C/O = 0.67 generated CNPs mostly in the form of primary particles with spherical-like shapes [64]. The PSD was bimodal, with the first mode between 2 and 4 nm and the second mode located around 8 nm [52]. For richer flames, CNPs in the form of aggregates with fractal shapes were more abundant, and the PSD continued to be bimodal; the first mode was located close to 2 nm, while the second mode was larger and reached values close to 70 nm for C/O = 0.77 and 90 nm for C/O = 0.82, respectively (Figure 2). There was no experimental data on PSD for C/O = 0.87; however, it is possible to assume that the PSD moved toward a slightly larger mode (above 100 nm), preserving the shape, as well known in the literature [65,66].

**Figure 3:**
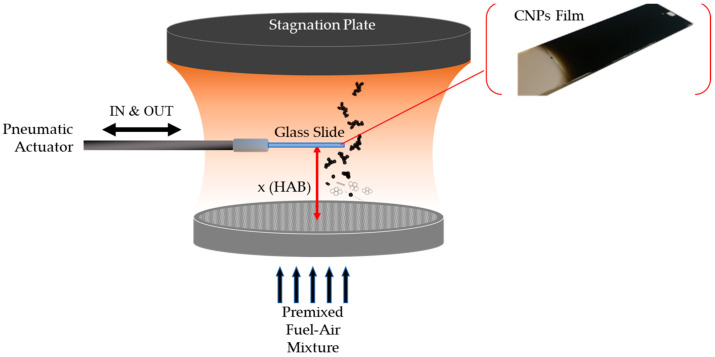
Scheme of the apparatus that deposits the CNPs on the glass slide in the McKenna burner. Cartoon showing the creation and growth of the CNPs along the flame’s height. Picture of a formed CNP film.

The CNPs were deposited on a rectangular glass slide (Aptaca microscope slides, 25.4 × 6.2 mm, 0.8/1 mm thick, hydrophilic, CA = 42.4 ± 3.9°), which iteratively went into the flame via rapid insertion thanks to a pneumatic actuator (Figure 3). The insertion time of the glass slide in the burner was 2 s and the rest time outside the burner was 10 s to minimize the substrate heating and always ensure a high temperature difference between the cold substrate (~350 K) and the hot flame (~1700 K) [63,67]. This high temperature difference allowed the harvesting of the nanoparticle via thermophoresis thanks to a local thermal gradient of the order of ~10^6^ K/m, close to the substrate [32,68]. The number of insertions, N, of the substrate in the flame was changed according to the C/O ratios and increased with geometric progression from 1 to 256. The C/O ratio and N were both varied to obtain films with different chemical and physical properties; by operating with C/O = 0.67, mostly primary spherical CNPs were deposited on the substrate, while at larger C/O ratios, the substrate was mostly exposed to CNP aggregates with fractal shapes (Figure 2).

The amount of deposited CNPs was evaluated in terms of the equivalent optical film thickness. An Agilent UV-Vis 8453 spectrophotometer and a 1-cm path-length quartz cuvette were used for measuring the UV-visible (UV-Vis) absorption spectra of CNPs films deposited on the glass slid from 190 nm to 950 nm [54,69]. Glass borosilicate absorbs light below 350 nm; hence the absorption spectra of the CNP films could only be evaluated above this wavelength. For all samples, we evaluated an equivalent optical thickness ${(\delta }_{s})\;$with the Beer-Lambert law, Equation (5), using the absorbance spectrum values, *ABS (λ)*:

(5) $$ABS(\lambda )=\frac{4\pi }{\lambda }k_{s}(\lambda {)\delta }_{s}$$

In particular, the value of λ = 532 nm was used to calculate $\delta_{s}$ since this is considered the standard wavelength for soot and, in general, for CNP analysis [65]. In Equation (5), k_s_ is the complex part of the soot refractive index, equal to 0.56. It is worth noticing that the thickness calculated in this way is not representative of the actual geometrical thickness of the film, which is composed of an arrangement of CNPs and voids, but rather represents the thickness of a layer of compact CNPs whose absorptions are equivalent to that of the film.

### 3.2. Contact Angle Measurement

**Figure 4:**
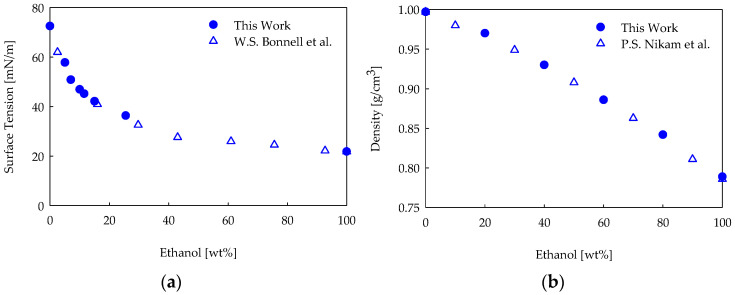
Surface tension (**a**) and density (**b**) as a function of the ethanol/water mixture composition. The filled circles are the values measured in this work and the empty triangles are the values from the literature [70,74].

The wettability tests of the CNP films were carried out with distilled water and distilled water/ethanol (≥99.8%, Honeywell, Seelze, Germany) mixtures. By increasing the ethanol concentration in water from 0 to 100 wt%, the surface tension, $\gamma_{LV}$, of the binary mixture decreased from ~72.8 mN/m to ~21.6 mN/m (Figure 4a) [70]. The mixture surface tensions were measured using the tensiometer FTA1000 (First Ten Angstroms Inc., Newark, CA, USA), using the pendant drop method before each wettability test session. The drop volume was dispensed very slowly with the motorized syringe of the FTA1000 and the pump rate was fixed at 0.4 μL/s for all the experiments. The surface tension was calculated by analyzing the shape of the pendant drop just before its detachment from the needle [71]. An image of the pendant drop was acquired using the software FTA32 Video 2.1 connected to the instrument to evaluate the surface area, the volume and the apex curvature of the drop, and, finally, to calculate the value of the surface tension. Liquids with a lower $\gamma_{LV}$ may tend to climb up the outer surface of the needle during pumping. To prevent this effect, the needle was rubbed with parafilm [72]. To calculate the surface tension with the pendant drop, the density difference between the liquid and air [71] must be known. The densities of the mixtures selected for the wettability tests were measured using a densimeter (DM 4500 M, Anton Paar, Graz, Austria) according to standard methodologies [73,74]. The density varied from 0.997 g/cm^3^ (pure water) to 0.789 g/cm^3^ (pure ethanol) as the mass percentage of ethanol in distilled water increased (Figure 4b). Figure 4a,b show data deriving from five replicates with excellent reproducibility obtained and error bars smaller than the symbol size.

The mixtures chosen for the CNP film wettability analysis were created using 5, 7, and 15 wt% ethanol in distilled water, leading to surface tensions of 58 mN/m, 51 mN/m, and 42 mN/m, respectively. The surface tensions were measured before each wettability test to ensure that the wetting liquid kept its properties intact. The corresponding densities for the three mass percentages of ethanol in water were 0.991 g/cm^3^, 0.998 g/cm^3^, and 0.976 g/cm^3^, respectively. It is worth noticing that preliminary tests with several mixture compositions were run to explore the wettability sensitivity to the liquid surface tensions of our samples. In this way, we individuated the surface tensions that were most appropriate to exploit the different wettability behaviors of the produced films, enhancing the differences among them.

The contact angles of the produced CNP coatings were measured using the tensiometer FTA1000 (First Ten Angstroms, Inc.) following the sessile drop method. The drop image was captured from the side view of the sample, and the contact angle was measured by using the software FTA32 Video 2.1. Depending on the surface wettability, different fitting equations were used: the Young-Laplace fit for contact angles characteristic of superphobic surfaces [75,76], and the spherical and the polynomial fit for contact angles characteristic of philic surfaces [40]. A 0.406 mm outer diameter needle was used for each test. The liquid was slowly pumped at a rate fixed at 0.4 μL/s [77]. Depending on the surface tension and density of the wetting liquid, a drop of a certain volume was formed before it fell under its own weight (e.g., for distilled water, the drop volume was 8 ± 0.1 μL). The distance between the needle tip and the surface was carefully evaluated to be minimal (~0.5 mm) to limit the experimental artifacts related to the impact and vibrations of the drop coming in contact with the sample’s surface. Each film was produced in many replicates and tested at several points with at least 15 drops; in this way, we checked the sample production reproducibility, and we monitored the surface coating uniformity [77]. The samples were analyzed just after their preparation and over time, in some cases, even after 1 year, and no appreciable changes in terms of wettability were detected.

## 4. Results

### 4.1. Properties of CNP Films

**Figure 5:**
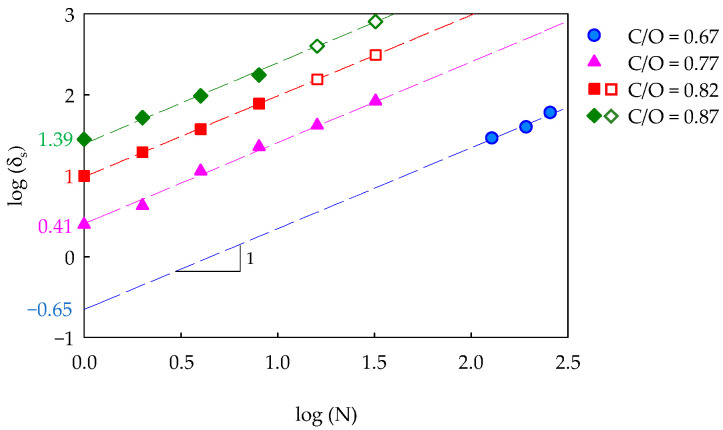
Equivalent optical thickness of CNP coatings on glass slides as a function of the number of insertions in the flame for different C/O ratios. The continuous lines are the fitting of the experimental points (filled symbols). The empty symbols indicate the equivalent optical thickness of the samples not measured with the UV-vis tests but estimated with the fitting lines.

Table 1 summarizes all the experimental conditions used in this work. For each condition, a picture of the coated substrate is reported to qualitatively highlight the similarities and differences among the samples. It is evident that the degree of blackening increases with the number of insertions or the C/O ratio. A quantitative analysis correlating the degree of blackening to the amount of deposited CNPs is here conducted through UV-vis measurements by evaluating the equivalent optical thickness, δ_s_, Equation (5). Figure 5 shows the δ_s_ of the CNP coatings at different C/O ratios as a function of the number of insertions.

Each set of data in Figure 5, graphed in terms of log(δ_s_) as a function of log(N), was interpolated with a line having a slope equal to one, thus indicating the linear increase in δ_s_ with N for all C/O ratios. In view of the observed linearity, the intercept of each fitting line allowed for estimating the coating growth rate, varying from 0.22 nm/insertion (C/O = 0.67) to 25.08 nm/insertion (C/O = 0.87). Clearly, the same equivalent optical thickness could be obtained with fewer flame insertions by increasing the C/O value. Finally, it is worth noticing that the values of δ_s_ of the samples at C/O = 0.82 and 0.87 obtained with 16 and 32 insertions could not be measured with the UV-vis spectrometer due to the very high blackening level. An estimate of δ_s_ for these conditions (plotted in Figure 5 with empty symbols) was identified by using the corresponding fitting lines. It is worth remembering that the equivalent optical thickness evaluated with Equation (5) considers the film as produced by the CNPs in the absence of void, so it can be correlated with the sole amount of deposited carbon nanoparticles without taking into account the specific topographic architecture of the coating. However, the linearity of the δ_s_ growth with N suggests that the void grade is preserved with film growth in a self-repeating structure.

### 4.2. Wettability with Distilled Water

**Figure 6:**
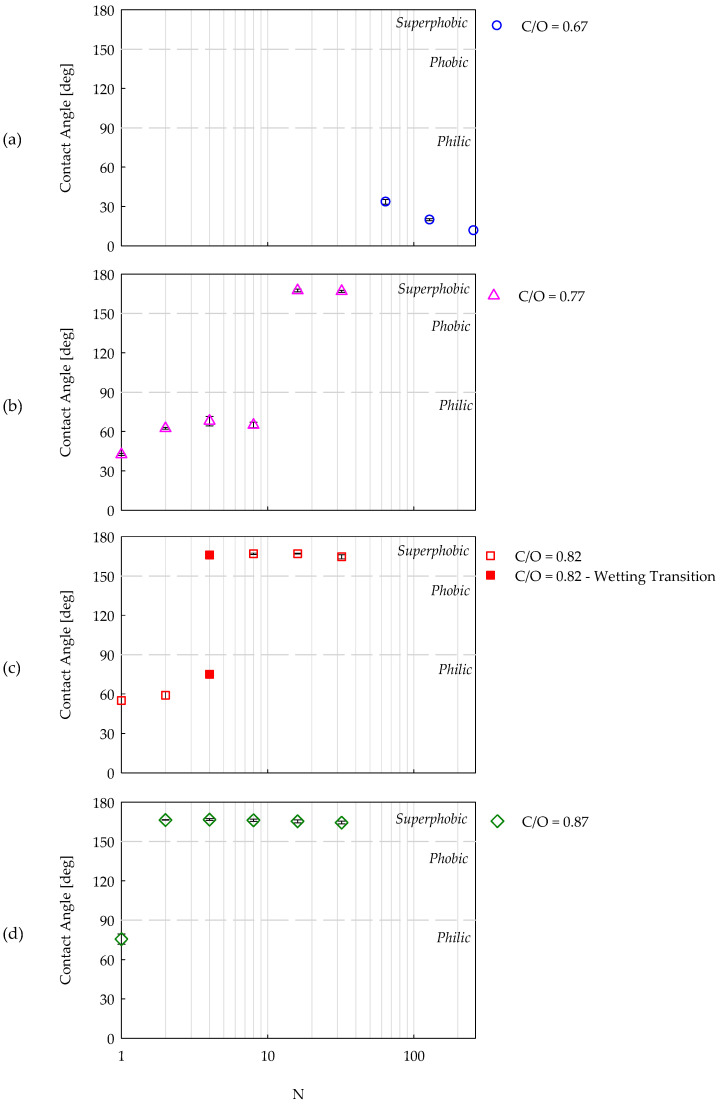
Distilled water contact angle as a function of the number of insertions (N) for CNP films produced with an ethylene-air flame with four C/O ratios equal to (**a**) 0.67, (**b**) 0.77, (**c**) 0.82 and (**d**) 0.87. The dotted lines delimitate the region of hydrophilic, hydrophobic, and superhydrophobic behavior.

Figure 6 shows the contact angle as a function of the number of insertions in a flame for the four different conditions examined in this work. Two different behaviors can be observed for the incipient sooting (C/O = 0.67) and fully sooting flames (C/O = 0.77, 0.82, and 0.87). In the former case, the samples were always hydrophilic, with a CA decreasing with the number of insertions. In the latter cases, the coatings passed from hydrophilic to superhydrophobic by increasing the number of insertions and, in particular, for higher C/O ratios, fewer insertions were required to obtain superhydrophobic surfaces (N = 16, 4, 2, for C/O = 0.77, 0.82, 0.87, respectively). Moreover, when hydrophilic, the samples had a contact angle that slightly increased with the number of insertions or the C/O ratio; when superhydrophobic, the samples had a constant CA equal to 166.2° with a standard deviation smaller than 1°, regardless of the flame conditions and the number of insertions. The experiments revealed that the passage from a hydrophilic to a superhydrophobic response of the film was not gradual but very sharp. For the sample produced with C/O = 0.82 and four flame insertions, two values of the CA were indicated in Figure 6c. This is due to the occurrence of a wetting transition; the sample was superhydrophobic upon deposing the drop, and after 0.1 s, a sudden decrease in CA to 72.2 ± 3.5° was observed.

### 4.3. Wettability with Ethanol/Water Mixtures

The measurement of the CA with liquids having surface tensions lower than that of water has the intent of a good wetting of the coating to better characterize these surfaces, extending the information on their morphology, and differentiating the nanofilms deriving from flames with different C/O values.

**Figure 7:**
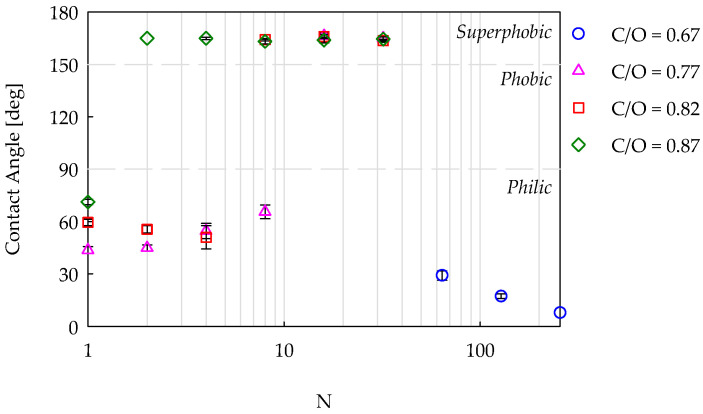
Contact angle as a function of the number of insertions in flames, with four different C/O ratios. Test liquid: a 5 wt% ethanol/water mixture with a surface tension equal to 58 mN/m.

Figure 7 shows the CAs obtained with drops of the 5 wt% ethanol/water mixture, producing a surface tension of 58 mN/m deposed on different coatings. The data are reported as a function of the number of insertions. For all the samples, the results are very similar to those observed with pure water, and in particular, it is worth mentioning that: (i) the value of the CA in the superphobic region was equal to 164.7 ± 1°, two degrees smaller than that measured with distilled water, (ii) the passage from philic to superphobic behavior occurred at the same number of insertions, (iii) the value of the CA in the philic region was slightly lower and consistent with the reduction in the liquid surface tension. The only qualitative difference was observed for the sample C/O = 0.82, N = 4. While this coating showed a wettability transition from superhydrophobic to hydrophilic behavior with water, it was now immediately philic.

**Figure 8:**
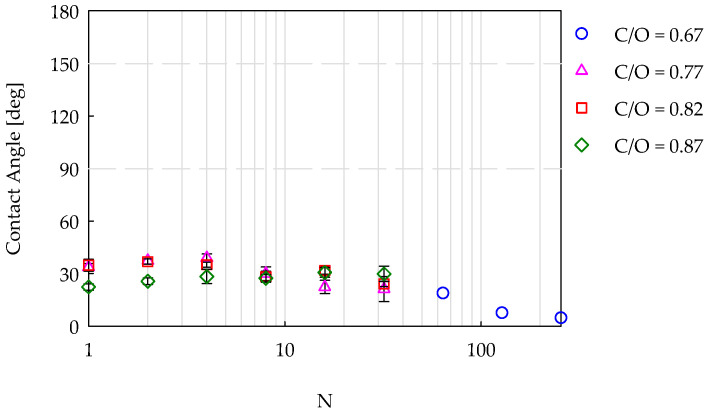
Contact angle as a function of the number of insertions in flames, with four different C/O ratios. Test liquid: a 15 wt% ethanol/water mixture with a surface tension equal to 42 mN/m.

Figure 8 shows the results obtained with the 15 wt% ethanol/water mixture, producing a surface tension of 42 mN/m. In this case, only the philic behavior was observed for all the samples, regardless of how they were produced with fully sooting (C/O = 0.77, 0.82, and 0.87) or incipient sooting (C/O = 0.67) flames. For the coatings deriving from fully sooting flames, the lowest values of the CA were measured for the samples previously showing superhydrophobicity. In regards to the samples deriving from C/O = 0.67, the values of the CA decreased with respect to those obtained for higher surface tensions (Figure 6a and Figure 7). Moreover, the coating obtained with 256 insertions had a CA ≤ 5°, indicating superphilic behavior.

### 4.4. Wettability vs. Film Thickness

**Figure 9:**
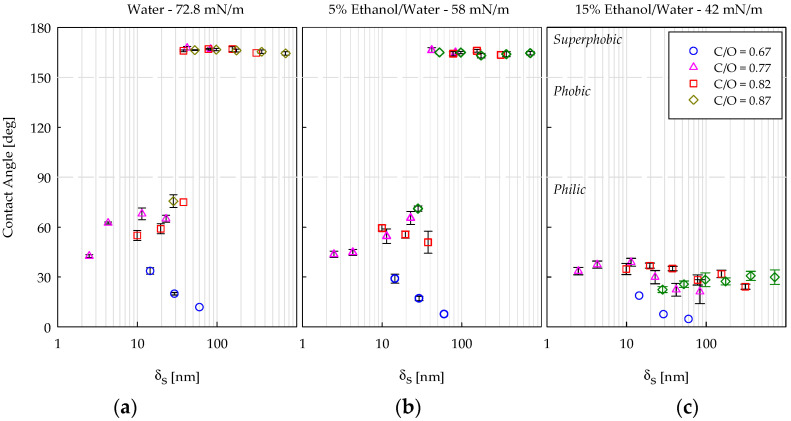
Contact angle as a function of the equivalent optical thickness of CNP films produced with an ethylene-air flame with four C/O ratios and using different liquid tests: (**a**) water, (**b**) 5% Ethanol/Water and (**c**) 15% Ethanol/Water.

The passage from philic to superphobic CA, observed in Figure 6 and 7, takes place in correspondence with a similar degree of blackening of the CNP coatings (Table 1). This suggests a different way of data plotting, i.e., plotting the contact angle as a function of the deposited CNP amount, i.e., as a function of the equivalent optical thickness of the samples, calculated with Equation (5). The results are shown in Figure 9, and except for C/O = 0.67, all data collapsed on a master curve for each surface tension of the wetting liquid. The data perfectly overlapped in the superphobic region, while they slightly scattered in the philic region. Figure 9a,b indicate that the sharp passage from philic to superphobic behavior occurs in correspondence with critical optical film thickness, roughly between ~30 and ~50 nm, i.e., a critical amount of deposited CNPs. This film thickness, necessary to obtain superphobic coatings, can be “built” with a heavy sooting flame (C/O = 0.87) by depositing many large CNPs (>100 nm) in few insertions or with C/O = 0.82 and 0.77 by depositing few small CNPs (<100 nm) in many insertions. Let us consider that superphobicity can be predicted with a Cassie-Baxter approach, thus, the passage from philic to superphobic behavior must depend on the film’s roughness. The existence of the master curves in Figure 9 thus suggests that the coatings at the philic/superphobic passage share the same thickness and roughness, regardless of the C/O ratio. For thin coatings with a thickness below ~30 nm (Figure 9a,b), the CAs with C/O = 0.77, 0.82, and 0.87 show small differences. In this case, we are probably in a hybrid wetting regime between Wenzel and Cassie-Baxter, with some coating areas completely wetted by the liquid, and others not-completely wetted, with some air pockets between solid surface and liquid already. Consequently, the CA values are more noisy because they are affected by surface irregularities [78,79]. Anyway, notwithstanding these discrepancies, data for the three C/O ratios were quite similar, and this suggests similar chemical-physical properties among these coatings.

In Figure 9c, there are the results with the 15 wt% ethanol/water mixture. In this case, the use of a low value of surface tension (42 mN/m) allows a good wetting of the coatings at all optical equivalent thickness values. It appears that the CA decreased around the equivalent optical thickness of approximately 30–50 nm, which is the same critical δ_s_ range detecting the passage from philic to superphobic behavior in Figure 9a,b. This confirms that, once wetted, the roughness allowing superphobic behavior with high surface tension liquids enhances wettability with low surface tension liquids. In addition, the CA reached a plateau at a high thickness, and this suggests the achievement of a coating with constant chemical properties and roughness, i.e., with “self-similar” topography.

For the incipient sooting flame, C/O = 0.67, superphobicity was never attained, even for film thicknesses exceeding ~50 nm. The value of the CA decreased with the thickness (Figure 9), thus indicating an increase in the coating’s roughness according to Equation (2).

The crucial difference between C/O = 0.67 and the other C/O ratios is in regard to the dimensions and shapes of the carbon nanoparticles deposed on the support. As already observed, in the incipient sooting flame, the CNPs are small (2–8 nm), and, according to the literature [80], they still have a roundish shape. In a more sooting flame, the CNPs form fractal aggregates with dimensions reaching 100 nm (Figure 2). This original fractal shape is a necessary condition to create a hierarchical roughness adequate to induce superphobicity. Our tests indicated that when the CNPs are still in their native spherical shape, they are not able to create, during the thermophoretic deposition, this hierarchical roughness and so, even if the coating has a sufficient thickness, it never shows superphobicity. In addition, the CA values measured at δ_s_ > 50 nm are higher for the fully sooting flame (~27°) compared to those from the incipient sooting flame (from 3° to 19°). Considering that the coatings produced with fractal CNPs are rougher than those created with spherical CNPs, the CA difference may suggest that they are not both in the Wenzel wetting regime or that they are chemically different. On CNPs produced at C/O = 0.67 we expect to find more oxygen atoms respect to the CNPs produced at larger C/O ratios [64] and this promotes wettability. Concerning the wetting regime, in hierarchical fractal geometries, the liquid can partially penetrate into the roughness, leading to a hybrid wetting regime between Wenzel and Cassie-Baxter.

### 4.5. Wettability Transitions

The sessile drop method used to estimate the wettability of a solid surface typically yields a value of the contact angle that remains the same from the beginning to the end of the test. A transition from higher to lower values passing from superphobic to philic behavior can also be observed [48,81]. All CA data shown in Figure 6–9 were constant in time except for the sample (Figure 6c) obtained with C/O = 0.82 and four insertions, producing an equivalent optical thickness of 38.8 nm. In this case, the CA was 166.4 ± 1° for the first 0.1 s of the test and then reduced to 72.2 ± 3.5°, thus indicating a wetting transition [49,82].

**Figure 10:**
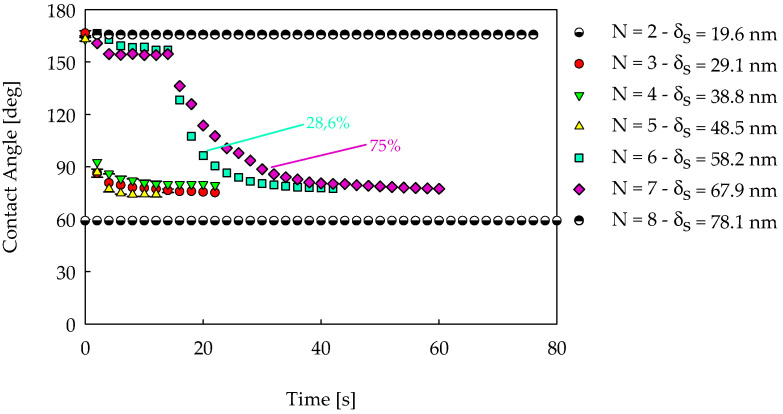
Time evolution of the CAs of coatings obtained with different numbers of insertions in a flame with C/O = 0.82. The percentage values are depicted via the cyan squares and the purple diamonds indicate the sample fraction area having the CA evolution graphed in the plot.

To conduct a more comprehensive analysis of the wetting transition, we focused on the flame with C/O = 0.82 by fabricating new coatings with the thickness changing from 19.6 nm to 77.6 nm obtained by progressively increasing the number of insertions from two to eight. It is worth remembering that previous results (Figure 6c) indicated that with two and eight insertions, no transitions were detected, and for N = 2 the behavior was hydrophilic, while for N = 8 it was superhydrophobic. These new tests were run with distilled water, and the results are shown in Figure 10 in terms of CA as a function of time. The sample produced with two insertions confirmed its stable hydrophilic response, reaching the value of 59° as soon as the water droplet touched the coating. The samples created with three, four, and five insertions shared a similar wetting transition behavior: they were superhydrophobic (CA = 161.6 ± 2.8°) for the very first instants after drop deposition and then transited to a hydrophilic state (CA = 75.7 ± 5°). The time during which the droplet maintained a spherical-like shape, denoted as stability time (τ), was less than 0.1 s. Figure 10 also shows that a stable hydrophilic contact angle is established after approximately 5 s. The sample obtained with six insertions had a wetting transition occurring with two different timings: 71.4% of the sample area had a very short stability time, τ < 3 s, while the complementary 28.6% showed significantly longer τ values of approximately 15 s. Still, in all cases, the final behavior was hydrophilic, with CA values passing from 166.6 ± 2° to 74.6° ± 5.6°. The sample produced with seven flame insertions also showed a transition with two different timings, but the area with a τ of approximately 15 s increased from 28.6% to 75% of the entire sample surface. The sample obtained with eight insertions, consistent with previous data, did not show any wetting transition, always maintaining a superhydrophobic Cassie-Baxter state. The water droplets on this coating maintained their shape and the CA value for approximately 1800 s (not reported in Figure 10), and during this time interval, they evaporated when passing from 8 μL to 2 μL. For smaller volumes, the accuracy of the CA measurement was low, and the tests were thus stopped.

The results in Figure 10 confirm that during the wettability tests, the thin coatings (N = 2, δ_s_ = 19.6 nm) were stably hydrophilic with CA = 59° and the thick coatings (N = 8, δ_s_ = 78.1 nm) were stably superhydrophobic with CA = 166°. In between, there were several samples showing wetting transitions from these two limit CAs, and the stability time tended to increase with the sample thickness. The extreme variability of these data is undoubtedly a sign of the inhomogeneity of the tested coating surface.

**Figure 11:**
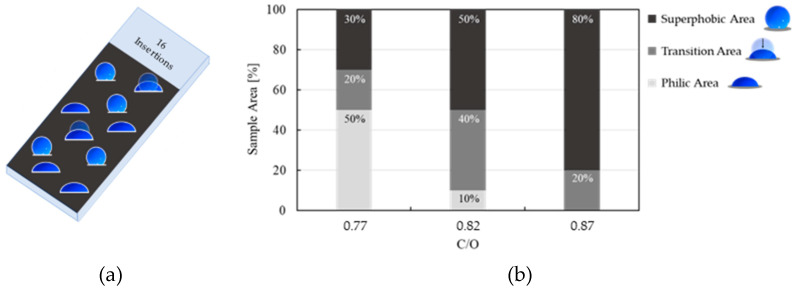
(**a**) Scheme representing a sample measured with the sessile drop method in different points showing philic, transition and superphobic areas and (**b**) bar graph reporting the samples’ percentage area having a philic transition (light grey), superphobic-philic transition (medium grey), and superphobic (dark grey) behavior tested with 7 wt% ethanol/water (51 mN/m) for different C/O ratios with 16 insertions.

To amplify the use of the wettability test in exploring surface homogeneity/inhomogeneity, liquids other than water can be exploited. Here, we focused our attention on selected samples obtained with sixteen insertions and three different flame conditions: C/O = 0.77, 0.82, and 0.87. These samples exhibited the same wettability in previous tests: they all were superphobic with water (72.8 mN/m, Figure 6) and a 5 wt% ethanol/water mixture (58 mN/m, Figure 7), and philic with a 15 wt% ethanol/water mixture (42 mN/m, Figure 8). We then compared them with ethanol/water mixtures with 7 wt% ethanol, producing an intermediate surface tension of 51 mN/m. With this liquid test, the wettability of each sample was variable, showing regions of philicity and superphobicity and a transition from superphobicity to philicity, as sketched in the cartoon in Figure 11a. The regions with different wettability were randomly distributed on the sample surface. Figure 11b shows bars indicating the percentage of the coating surface with philic behavior, superphobic behavior, and the superphobic-philic transition. The samples produced with C/O = 0.77 were mainly philic. By increasing the flame C/O ratio, the philic regions progressively reduced with increments in the superphobic zones.

## 5. Conclusions

The production of flame-generated CNP coatings is a promising process that, under suitable tuning of the combustion and the deposition parameters, is able to generate thin films with customized wettability, deriving from a favorable combination of coating chemistry and morphology. Here, wettability tests are adopted to assess both the wettability behavior of CNP coatings and to quickly monitor their non-uniform roughness and topographical irregularities.

The CNP coatings were studied for different combustion conditions and film thickness. The CNPs were produced in a rich premixed ethylene/air flame with C/O ratios equal to 0.67, 0.77, 0.82, and 0.87. These four conditions led to the production of different CNPs, ranging from almost spherical particles smaller than 8 nm (C/O = 0.67) to fractal aggregates larger than 100 nm (C/O = 0.87) [53,54,80,81,82,83]. Wettability tests were run by measuring the contact angle, CA, of the coatings with the sessile drop method. The liquids used for the tests included pure water and ethanol/water mixtures, enabling a reduction in the liquid surface tension from 72.8 mN/m to 42 mN/m. The wettability analysis indicated the existence of two distinct types of coatings.

The CNP coatings produced in the incipient sooting flame (C/O = 0.67) always exhibited philic behavior with water and even more with ethanol/water mixtures. In particular, the CA decreased by increasing the film thickness or reducing the liquid surface tension. A thickness increase led to an increase in roughness, which, in accordance with the Wenzel model (Equation (2)), promoted better wetting of the surface.

For fully sooting flames (C/O = 0.77, 0.82, and 0.87) where the CNPs had a fractal morphology, the thin coatings (δ_s_ < ~30 nm) were hydrophilic, while the thick coatings (δ_s_ > ~50 nm) were superhydrophobic with a critical δ_s_ ranging from ~30 to ~50 nm. It is important to note that δ_s_ results are the main feature of the coatings, regardless of the effective original size of the CNP fractal aggregates. The similarity of coatings deriving from fully sooting flames was confirmed via the wettability tests with the lowest surface tension liquid (42 mN/m). In this case, all the samples were philic, and the CA, again, only depended on δ_s_. In particular, for δ_s_ > ~50 nm, the CA reached a philic plateau, and this suggests that above the critical thickness, the coating grows, maintaining constant chemical properties and roughness morphology. For intermediate thickness from ~30 to ~50 nm, an extreme variability of wetting behavior was observed, as also highlighted with the scatter of CA data in this region (Figure 9 and Figure 10). This result highlights the surface heterogeneity of flame-produced coatings. It should be underlined that these non-uniformities are also present for thick, stably superhydrophobic films, as shown with a lower surface tension fluid (51 mN/m).

Overall, this paper clearly indicates the possibility of producing thin films with CNPs, tuning the surface properties by controlling particle size/morphology and film thickness. This opens the possibility for future studies on CNP applications in biological and sensing applications where the surface properties are key parameters to control. Finally, the results demonstrate that wettability analysis is a simple and rapid tool to analyze surface topography and homogeneity/heterogeneity. Further studies are needed to provide detailed correlations between film morphology and wettability data.

## Figures and Tables

**Table 1:** Photos of CNP coatings obtained with different C/O ratios and the number of sample insertions in the flame.

		Insertion Number in the Burner								
		1	2	4	8	16	32	64	128	256
C/O	0.67							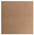	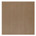	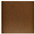
	0.77	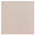	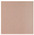	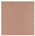	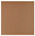	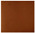	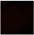			
	0.82	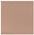	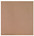	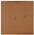	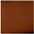	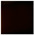	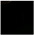			
	0.87	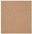	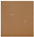	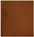	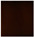	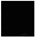	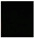			

## Data Availability

The data presented in this study are available upon request from the corresponding author.
